# Impact of Glycemic Control on Coronary Inflammation Evaluated by Computed Tomography Pericoronary Fat Attenuation Index in Patients with Acute Coronary Syndrome

**DOI:** 10.31083/j.rcm2407203

**Published:** 2023-07-14

**Authors:** Jinyao Jiang, Yan Yin, Yilin Li, Bihe Xu, Zhiguo Zou, Song Ding, Jun Pu

**Affiliations:** ^1^Department of Cardiology, Renji Hospital, Shanghai Jiao Tong University School of Medicine, 200127 Shanghai, China; ^2^Department of Radiology, Renji Hospital, Shanghai Jiao Tong University School of Medicine, 200127 Shanghai, China

**Keywords:** acute coronary syndrome, coronary inflammation, coronary computed tomography angiography, diabetes mellitus, fat attenuation index

## Abstract

**Background:**

Coronary inflammation causes significantly increased risk of cardiovascular disease (CVD) in diabetic patients. This study investigated the relationship between coronary local inflammation, detected by pericoronary fat attenuation index (FAI), and different blood glucose control levels in low-risk acute coronary syndrome (ACS) patients with or without diabetes.

**Methods:**

A total of 309 patients with low-risk ACS were classified into three groups: non-diabetes, well-regulated diabetes, and poorly regulated diabetes. Pericoronary FAI around the proximal or left anterior descending artery (LAD), left circumflex artery (LCX), and right coronary artery (RCA), were evaluated by coronary computed tomography angiography (CCTA), and systemic inflammatory variables and other biochemical indicators were detected by flow cytometry.

**Results:**

Pericoronary FAI values around the proximal LAD, LCX, and RCA in poorly regulated diabetes were significantly higher than those in well-regulated diabetes and non-diabetes, whereas those in well-regulated diabetes were not statistically different from those in non-diabetes. Further, plasma glycated hemoglobin (HbA1c) level was positively correlated with the pericoronary FAI values in LAD, LCX, and RCA. However, no significantly increased systemic inflammatory mediators were found in diabetic patients with poor glycemic control.

**Conclusions:**

Diabetic patients with poor glycemic control may have higher coronary local inflammation as detected by pericoronary FAI surrounding the three major coronary arteries.

**Clinical Trial Registration:**

NCT05590858.

## 1. Introduction

Diabetes mellitus (DM) is a serious, chronic, metabolic disease characterized by chronic hyperglycemia, which affects approximately 10% of the global population and is expected to increase in prevalence [1]. The leading cause of mortality in the diabetic population remains cardiovascular disease, which is estimated to have a two-to-three-times higher risk than in individuals without diabetes [2, 3].

Poor glycemic control, defined by glycated hemoglobin (HbA1c) >7% [4], has been widely documented to be associated with a higher risk of cardiovascular disease (CVD) among diabetic individuals [2]. Several studies have demonstrated that intensive glucose control in diabetic patients could reduce the incidence of cardiovascular complications more than standard glucose control [5, 6, 7, 8, 9].

Vascular inflammation can be a cause of the significantly increased risk of CVD in diabetic patients [10, 11, 12]. Recent data have suggested that inflammation could cause metabolic defects in diabetes leading to endothelial injury and development of vascular complications [12]. Furthermore, chronic inflammation contributes to the development of coronary atherosclerosis and is one of the features of vulnerable coronary plaques [13]. Until recently, inflammation-assessment tools could not effectively evaluate coronary local inflammation. For example, systemic plasma biomarkers such as high sensitivity C-response protein (hsCRP) and pro-inflammatory cytokines are not directly related to the process of atherogenesis. Positron emission tomography (PET) imaging, as the gold standard in evaluating perivascular adipose tissue (PVAT) inflammation [14], is limited by its high cost, high exposure, and low clinical availability. Therefore, finding an accessible clinical detection method that reflects the current status of vascular inflammation is very important for the early diagnosis of cardiovascular complications. Fortunately, pericoronary fat attenuation index (FAI), derived from coronary computed tomography angiography (CCTA), has emerged as a novel imaging biomarker that overcomes these limitations and noninvasively detects coronary artery local inflammation. During active vessel inflammation, the paracrine inflammatory signals secreted by vascular walls would diffuse to PVAT and prevent local adipogenesis by affecting biological processes such as adipocyte proliferation, differentiation, and lipolysis. These lead to a change of composition of PVAT around inflamed arteries. This change could be captured by CCTA and presented as an attenuation that shifts from the lipid phase (more negative Hounsfield units [HU] values) to the aqueous phase (less negative HU values), known as the pericoronary FAI [15]. Our previous study demonstrated that pericoronary FAI is a useful imaging biomarker that helps identify vulnerable plaque characteristics [16]. Furthermore, a recent meta-analysis elucidated the role of pericoronary FAI in discriminating between stable and unstable plaques, adding information to the prognosis for future major adverse cardiovascular events (MACE) [17]. However, the previous study did not focus on the effect of glycemic control on pericoronary FAI, which is also of clinical importance in monitoring coronary inflammation progression and preventing CVD development.

Our present study aimed to clarify the relationship between blood glucose control and FAI-based pericoronary inflammation in low-risk ACS patients with and without diabetes. We hypothesized that pericoronary FAI might be a potential imaging biomarker that can reflect the inflammatory status associated with different blood-glucose-control levels.

## 2. Methods

### 2.1 Study Sample

This study retrospectively enrolled low-risk ACS patients who underwent CCTA examination before elective coronary angiography between January 2019 and December 2020 at Renji Hospital. The definition of low-risk ACS in the present study was that patients exhibited chest pain with or without electrocardiogram (ECG) ST-T changes, but no persistent ST-segment elevation, who were suspected of non-ST elevation (NSTE)-ACS, but did not conform to an immediate (<2 hours) or early (<24 hours) invasive strategy according to guidelines [18].

The inclusion criteria were: (1) patients exhibited chest pain but were troponin negative and were suspected of low-risk ACS; (2) patients underwent CCTA examination before elective coronary angiography; (3) patients with at least one significant stenosis (≥50%) in major epicardial vessels based on coronary angiography. Patients who were previously diagnosed with diabetes and were undergoing medical or lifestyle interventions, or met the diagnostic criteria according to the American Diabetes Association [19], were regarded as having diabetes.

**Fig. 1.**
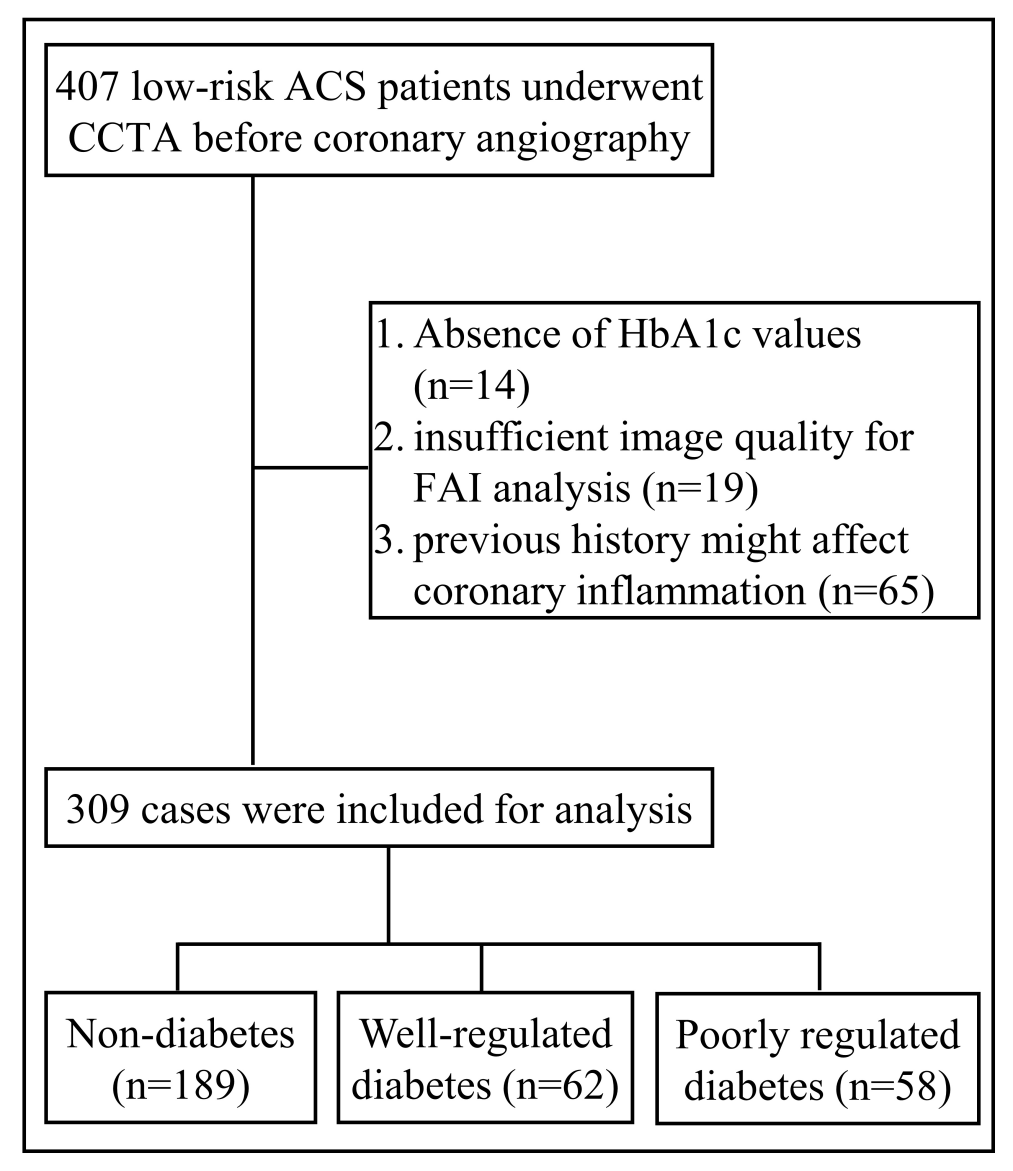
**Flow chart of study sample**. ACS, acute coronary syndrome; CCTA, coronary computed tomography angiography; FAI, fat attenuation index; HbA1c, plasma glycated hemoglobin.

The exclusion criteria were: (1) patients with missing preprocedural HbA1c values; (2) insufficient image quality for FAI analysis; (3) previous history of coronary revascularization or myocardial infarction; (4) chronic kidney disease requiring hemodialysis; or (5) malignant tumor, immune system disorders, or statin use within 3 months. A total of 309 patients were ultimately enrolled in the present study (Fig. 1). The baseline features for the study subjects were documented. This study was performed with approval from the Institutional Review Board (IRB) of Renji Hospital; written informed consent was waived as the current study was considered a retrospective review of anonymized clinical data.

### 2.2 CCTA Protocol and CCTA-Based FAI Analysis

CCTA examinations were performed using a 128-slice multidetector computed tomography (CT) (Aquilion ONE, Toshiba Medical Systems Corporation, Tokyo, Japan). To achieve optimal imaging quality, 25–75 mg oral metoprolol was administered prior to the examination to patients with heart rate >75 beats/min. An 80-mL bolus of contrast media was injected through the antecubital vein at an infusion rate of 5 mL/s followed by a 30-mL saline flush at the same speed. ECGs were used for retrospective gating to allow synchrony with the heartbeat. The imaging data were reconstructed at a 0.5-mm slice thickness and a 0.25-mm reconstruction interval.

**Fig. 2.**
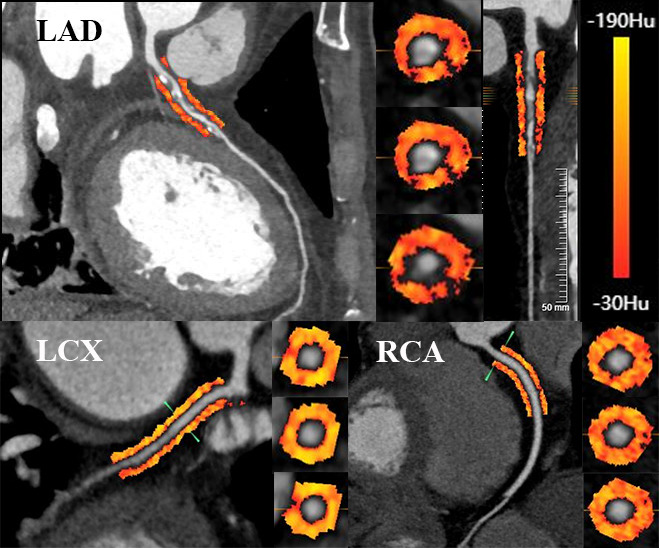
**Example of the color-coded quantitative analysis of pericoronary FAI surrounding the proximal LAD, LCX, and RCA**. FAI, fat attenuation index; LAD, left anterior descending artery; LCX, left circumflex artery; RCA, right coronary artery; HU, Hounsfield unit.

All reconstructed CCTA data were transferred to semi-automated post-processing software (United Imaging Intelligence, version R001, United Imaging Healthcare Co., Shanghai, China) for pericoronary FAI analysis. According to the landmark study by Antonopoulos *et al*. [15], pericoronary FAI was defined as the mean CT attenuation of coronary PVAT from –190 to –30 HUs, and coronary PVAT was defined as the adipose tissue located adjoining the coronary artery at a distance equal to the diameter of the vessel. To measure pericoronary FAI, the PVAT located in the proximal 40-mm segments of three major coronary arteries (LAD, LCX, and RCA), were traced and analyzed as previously described [20]. Notably, for LAD and LCX, the proximal 40-mm segments were analyzed, while for RCA, the proximal 10- to 50-mm segments were analyzed to avoid the interference of aortic wall on the most proximal 10-mm segments [20]. An example of pericoronary FAI analysis is shown in Fig. 2. To evaluate the reproducibility, FAI values were analyzed by two experienced radiologists who were blind to clinical data.

### 2.3 Measurement od Serum Inflammatory Cytokines and Other Biochemical Indicators 

Serum inflammatory cytokine (including interleukin [IL]-2, IL-4, IL-6, and IL-10) levels were measured using flow cytometry. Briefly, venous blood (4 mL) was collected into ethylene diamine tetraacetic acid (EDTA)-containing tubes at admission and centrifuged at 3000 g for 5 min. Serum was immediately separated, and ${\mathrm{BD}}^{\text{TM}}$ (Becton, Dickinson and Company, Franklin Lakes, NJ, USA) Cytometric Bead Array (CBA) kits were used for cytokine quantification according to the manufacturer’s instructions. Other biochemical indicators, including hsCRP, creatine kinase-MB (CK-MB), alanine transaminase (ALT), serum creatinine (Scr), and lipid parameters, were assayed at the time of hospital admission as well.

### 2.4 Statistical Analyses

Continuous variables were assessed by the Kolmogorov-Smirnov test for normality and were presented as means ± standard deviation (SD) when normally distributed or medians and interquartile range (IQR) when not normally distributed, while categorical variables were expressed as numbers and percentages. One-way ANOVA with Bonferroni *post hoc* tests were used to compare continuous variables; $\chi^{2}$ tests were used to compare categorical variables. Pearson correlational analysis was performed to analyze the associations between HbA1c and other variables, as appropriate. Interobserver variability of FAI values was assessed using intraclass correlation coefficient (ICC). Values of *p* < 0.05 were considered statistically significant. Statistical analyses were performed using SPSS (IBM SPSS 23.0, SPSS Inc. IBM Corp., Armonk, NY, USA).

## 3. Results

### 3.1 Clinical Characteristics

A total of 407 low-risk ACS patients underwent CCTA evaluation before elective coronary angiography in Renji Hospital. Catheterization recipients between January 2019 and December 2020 were screened. Patients were excluded due to absence of HbA1c values (*n* = 14), insufficient image quality for FAI analysis (*n* = 19), or previous history mentioned above (*n* = 65). The remaining 309 patients were finally enrolled and classified into three groups: non-diabetes, well-regulated diabetes, and poorly regulated diabetes, according to the presence or absence of diabetes and the glycemic control evaluated based on a target HbA1c value of 7% (see Figs. 1,2). Table 1 summarized the comorbidities and laboratory data at baseline, among these three groups, as well as other variables analyzed. None of the variables showed a significant difference between the diabetic and non-diabetic groups.

**Table 1.** **Clinical characteristics of the included participants by diabetes status**.

Variables		Participants without DM (n = 189)	Participants with DM		*p* value
			HbA1c ≤7.0 (n = 62)	HbA1c >7.0 (n = 58)	
Baseline characteristic					
	Age (years)	65.00 (60.00, 69.00)	67.50 (61.75, 73.00)	67.00 (61.00, 71.75)	0.378
	Sex males, n (%)	126 (66.7)	42 (67.7)	43 (74.1)	0.561
	Smoking, n (%)	64 (33.9)	18 (29.0)	20 (34.5)	0.755
	BMI (kg/$m^{2}$)	24.22 ± 3.07	24.38 ± 3.01	24.99 ± 3.15	0.248
	SBP (mmHg)	133.80 ± 18.86	134.16 ± 14.16	140.17 ± 17.88	0.055
	DBP (mmHg)	78.70 ± 9.85	76.40 ± 10.68	78.84 ± 9.76	0.261
	Hypertension, n (%)	113 (59.8)	40 (64.5)	41 (70.7)	0.308
	Heart rate (beats/min)	72.00 (66.00, 80.00)	73.50 (65.75, 83.00)	76.00 (65.00, 89.00)	0.134
Lipid profile					
	Triglycerides (mmol/L)	1.30 (0.94, 1.80)	1.24 (0.92, 1.70)	1.43 (1.18, 1.82)	0.508
	LDL cholesterol (mmol/L)	2.03 (1.64, 3.36)	1.85 (1.39, 2.26)	2.34 (1.65, 2.89)	0.064
	HDL cholesterol (mmol/L)	1.06 (0.91, 1.26)	1.03 (0.90, 1.17)	1.03 (0.88, 1.20)	0.114
	Dyslipidemia, n (%)	52 (27.5)	18 (29.0)	20 (34.5)	0.593
Biochemical findings					
	ALT (U/L)	20.00 (15.00, 27.00)	21.00 (15.00, 29.25)	19.50 (16.00, 27.00)	0.830
	Creatinine (µmol/L)	66.00 (56.00, 78.00)	68.00 (58.00, 78.00)	62.00 (56.00, 78.00)	0.911
	CK-MB (ng/mL)	1.80 (1.50, 6.58)	1.70 (1.10, 2.70)	2.00 (1.30, 3.20)	0.678
	NT-ProBNP (pg/mL)	296.26 (30.99, 502.00)	323.17 (72.27, 522.87)	366.18 (131.68, 603.95)	0.056
Medications					
	Insulin, n (%)	/	10 (16.1)	17 (29.3)	0.084
	OHA alone, n (%)	/	28 (45.2)	33 (56.9)	0.199

Continuous variables were presented as mean ± SD or median (IQR). Categorical variables were presented as number (percentage). BMI, body mass index; SBP, systolic Blood Pressure; DBP, diastolic Blood Pressure; LDL, low density lipoprotein; HDL, high density lipoprotein; ALT, alanine aminotransferase; CK-MB, creatine kinase-MB; NT-ProBNP, N-terminal pro brain natriuretic peptide; OHA, oral hypoglycemic agents; HbA1c, plasma glycated hemoglobin; DM, diabetes mellitus; IQR, interquartile range.

### 3.2 Pericoronary FAI Values and Blood Glucose Control Levels

**Fig. 3.**
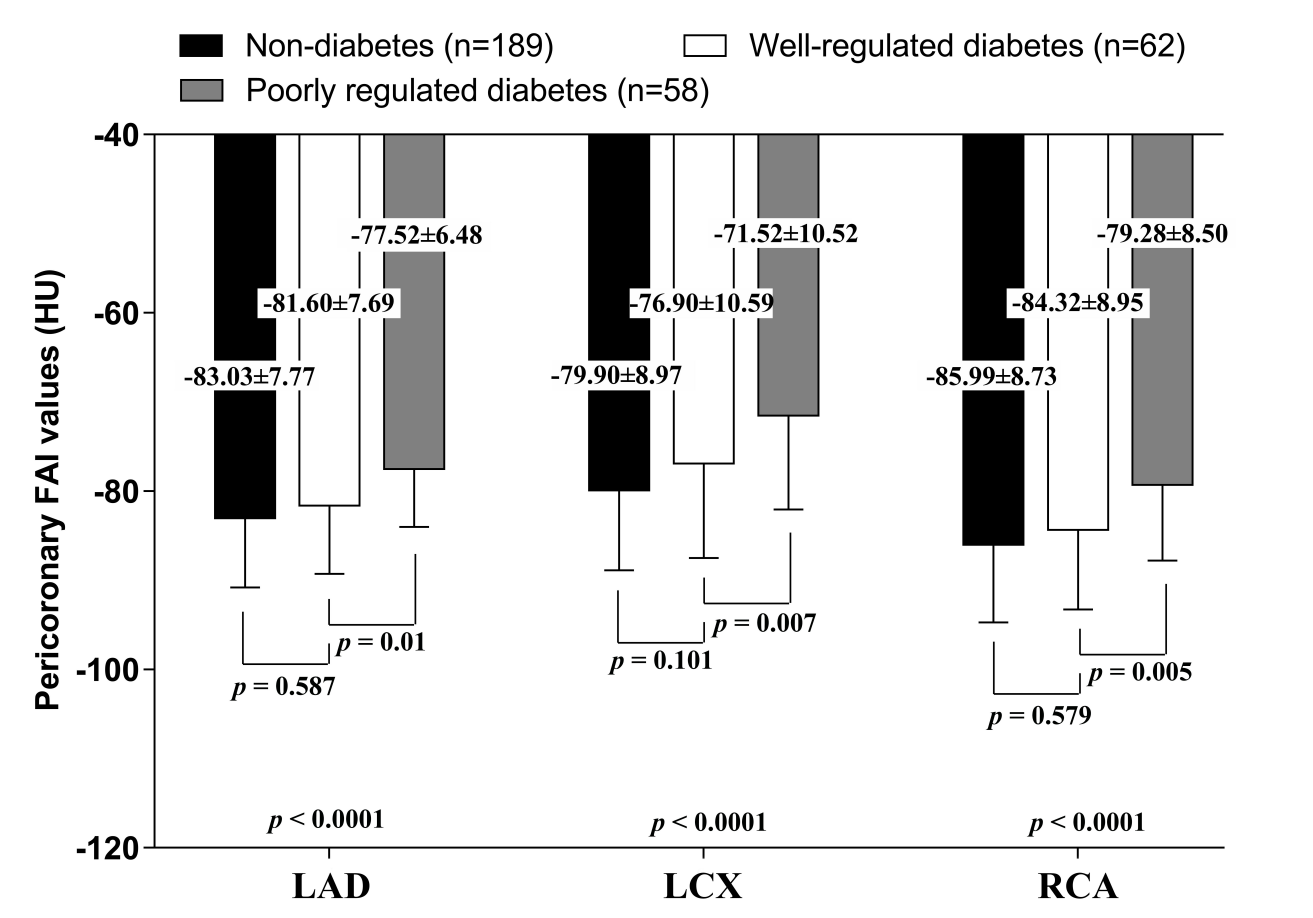
**FAI values around LAD, LCX, and RCA, among included participants by diabetes status**. FAI, fat attenuation index; HU, Hounsfield unit; LAD, left anterior descending artery; LCX, left circumflex artery; RCA, right coronary artery.

Fig. 3 shows the pericoronary FAI values around LAD, LCX, and RCA, among subjects with different glycemic statuses. The FAI values around the proximal LAD, LCX, and RCA in patients with diabetes were –77.52 ± 6.48 HU, –71.52 ± 10.52 HU, –79.28 ± 8.50 HU in those with poorly regulated glycemic values, and –81.60 ± 7.69 HU, –76.90 ± 10.59 HU, –84.32 ± 8.95 HU in those with well-regulated glycemic values. In patients without diabetes, it was –83.03 ± 7.77 HU, –79.90 ± 8.97 HU, –85.99 ± 8.73 HU. The FAI values around the proximal LAD, LCX and RCA in poorly regulated diabetic patients were significantly higher than those in well-regulated diabetic and non-diabetic patients (*p* < 0.05 in LAD, LCX and RCA), whereas those values in well-regulated diabetic patients was not statistically different with those in non-diabetic patients, although it was nominally higher as well (*p* = 0.413 in LAD, *p* = 0.165 in LCX, and *p* = 0.733 in RCA). Overall, the FAI values were significantly higher in patients with poorly regulated diabetes than in those with non-diabetes or well-regulated diabetes. There were no significant differences in FAI values between patients with well-regulated diabetes and those without diabetes.

### 3.3 Correlation of HbA1c Levels with Pericoronary FAI Values

**Fig. 4.**
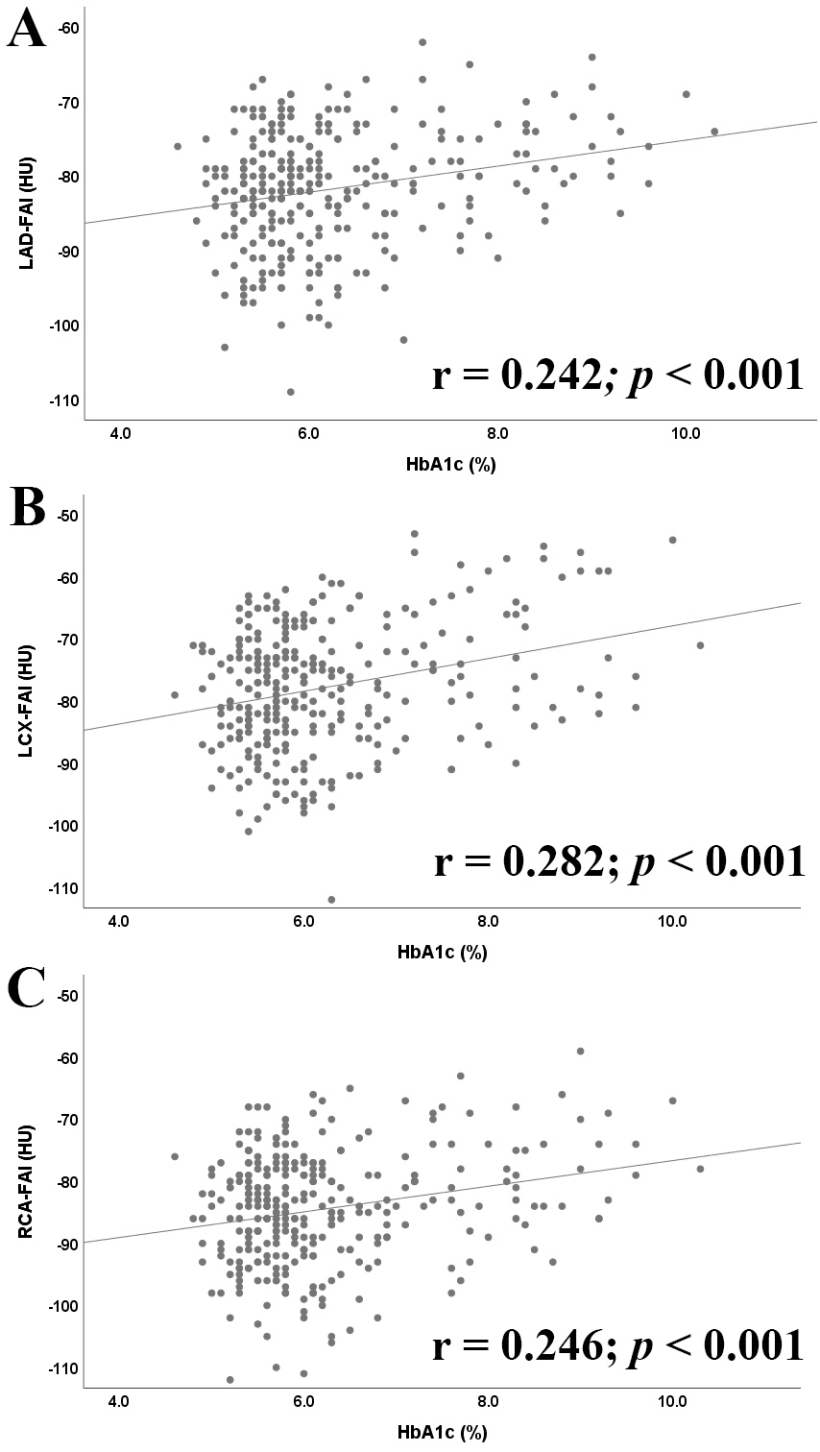
**Correlation between HbA1c Level and the FAI values around LAD (A), LCX (B), and RCA (C)**. FAI, fat attenuation index; HU, Hounsfield unit; LAD, left anterior descending artery; LCX, left circumflex artery; RCA, right coronary artery; HbA1c, plasma glycated hemoglobin.

In Fig. 4, the Pearson correlational analysis showed that there was a significant positive correlation of HbA1c level with the pericoronary FAI values whether in LAD (Pearson’s r = 0.242, *p* < 0.001), LCX (r = 0.282, *p* < 0.001), or RCA (r = 0.246, *p* < 0.001).

### 3.4 Glycemic Control and Relationship with Systemic Inflammatory Variables

We next explored whether the change in pericoronary FAI could be attributed to systemic inflammatory activation. As presented in Table 2, diabetic patients with poor glycemic control had nominally higher serum level of hsCRP. However, results did not show significant differences among the three groups (*p* = 0.635). Further, pro-inflammatory cytokines IL-2, IL-6 and anti-inflammatory cytokines IL-4, IL-10 were measured. Likewise, results were not statistically different among the three groups, although IL-6 was nominally higher in patients with poor glycemic control (*p* = 0.321).

**Table 2.** **Systemic inflammatory variables of the included participants by diabetes status**.

Variables		Participants without DM (n = 189)	Participants with DM		*p* value
			HbA1c ≤7.0 (n = 62)	HbA1c >7.0 (n = 58)	
hsCRP, mg/L		0.55 (0.50, 1.77)	0.80 (0.50, 1.26)	1.14 (0.52, 2.79)	0.630
Cytokine levels					
	IL-2, pg/mL	0.91 (0.50, 1.37)	0.94 (0.64, 1.31)	1.04 (0.79, 1.37)	0.925
	IL-4, pg/mL	1.04 (0.57, 1.77)	1.17 (0.55, 1.91)	0.92 (0.64, 1.57)	0.523
	IL-6, pg/mL	4.02 (2.59, 6.77)	4.30 (2.63, 6.92)	4.76 (3.30, 8.35)	0.321
	IL-10, pg/mL	1.63 (1.24, 2.29)	1.51 (1.23, 2.47)	1.67 (1.20, 2.52)	0.872

Continuous variables were presented as median (IQR). hsCRP, high sensitivity C-response protein; DM, diabetes mellitus; HbA1c, plasma glycated hemoglobin; IQR, interquartile range; IL, interleukin.

### 3.5 Impact of Duration of Diabetes on the FAI Values Around LAD, LCX, and RCA

Long-term diabetic duration was closely associated with impairment of coronary atherosclerosis. We next explored the impact of duration of diabetes on FAI values. As shown in Tables 3,4, diabetic patients with ≥10 years duration seemed to have higher FAI values around LAD, LCX, and RCA, than did those with <10 years duration (*p* < 0.05 in LAD, LCX and RCA). But in patients with well-regulated DM, the FAI values were not significantly different in patients with ≥10 years and <10 years duration (*p* > 0.05).

**Table 3.** **FAI values in relation to diabetes duration in patients with DM**.

FAI values	Patients with DM		*p* value
	<10 years (n = 54)	≥10 years (n = 66)	
LAD	–81.24 ± 7.748	–78.30 ± 6.866	0.030
LCX	–76.44 ± 10.507	–72.55 ± 10.894	0.049
RCA	–83.91 ± 8.945	–80.23 ± 8.878	0.026

Values were presented as mean ± SD. FAI, fat attenuation index; DM, diabetes mellitus; LAD, left anterior descending artery; LCX, left circumflex artery; RCA, right coronary artery.

**Table 4.** **FAI values in relation to diabetes duration in patients with well-regulated DM**.

FAI values	Patients with DM HbA1c ≤7.0		*p* value
	<10 years (n = 31)	≥10 years (n = 31)	
LAD	–82.84 ± 7.967	–80.35 ± 7.319	0.206
LCX	–78.52 ± 9.660	–75.29 ± 11.379	0.234
RCA	–85.00 ± 9.335	–83.65 ± 8.651	0.556

Values were presented as mean ± SD. FAI, fat attenuation index; DM, diabetes mellitus; LAD, left anterior descending artery; LCX, left circumflex artery; RCA, right coronary artery; HbA1c, plasma glycated hemoglobin.

### 3.6 Reproducibility

Interobserver variability of FAI values was strong for three coronary artery vessels and is shown in Table 5.

**Table 5.** **ICCs for interobserver variability for FAI values**.

FAI values	Interobserver variability	
	ICC	95% confidence interval
LAD	0.959	0.914−0.981
LCX	0.989	0.976−0.995
RCA	0.984	0.966−0.992

ICC, intraclass correlation coefficient; FAI, fat attenuation index; LAD, left anterior descending artery; LCX, left circumflex artery; RCA, right coronary artery.

## 4. Discussion

**Fig. 5.**
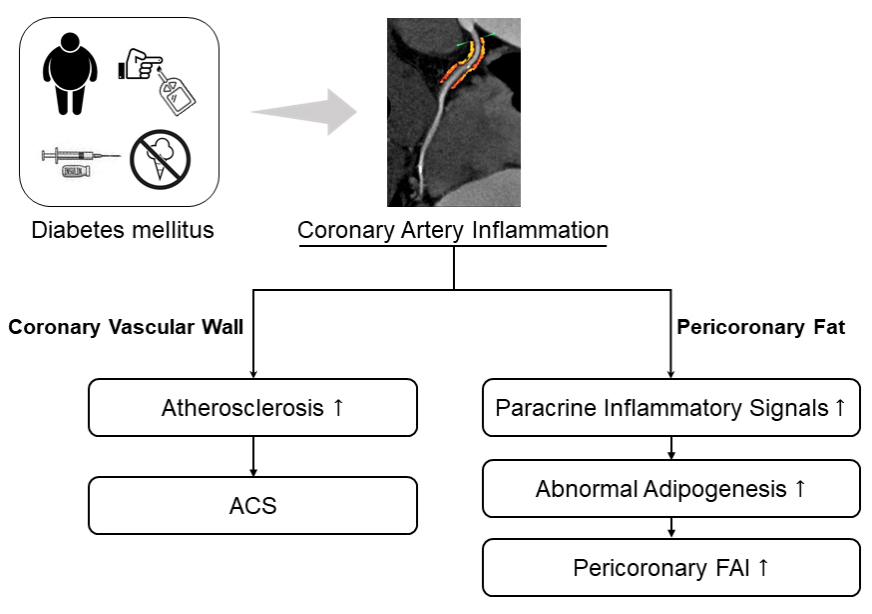
**Impact of glycemic control on coronary inflammation evaluated by pericoronary FAI in patients with acute coronary syndrome**. ACS, acute coronary syndrome; FAI, fat attenuation index.

We retrospectively investigated the association between diabetes status and a coronary artery local inflammation imaging biomarker, pericoronary FAI. The major finding of the current study was that pericoronary FAI was associated with glycemic control. Whether in LAD, LCX, or RCA, poorly regulated diabetic patients had higher pericoronary FAI than did either well-regulated diabetic patients or non-diabetic patients. Well-regulated diabetic patients had nominally higher perivascular FAI than did non-diabetic patients, although the difference was not statistically significant. Further, we found that the activation of coronary local inflammation with higher pericoronary FAI values in poorly regulated diabetic patients did not appear to be directly associated with systemic inflammation level, although the serum pro-inflammatory variables showed a nominal increase. These results potentially support the idea that poor glycemic control could activate coronary artery local inflammation and that this effect can be detected by pericoronary FAI analysis (Fig. 5).

ACS is an “inflammatory condition” for coronaries. The accumulating evidence has implicated an inflammatory process in the pathogenesis of ACS that involves local immune cells in coronary arteries generating inflammatory factors that promote thrombus formation [21]. Atherosclerosis has long been a crucial process of ACS, and vascular inflammation is considered to be a key feature in atherogenesis and atherosclerotic plaque rupture, leading to major cardiovascular events [11, 22, 23]. Many cardiovascular risk factors, including diabetes, contribute to this pathogenesis; reports showed that poor glycemic control yielded an impaired homeostasis of the metabolic environment, characterized by chronic inflammation, impaired fibrinolysis, oxidative stress, and increased expression of pro-inflammatory cytokines, which aggravate the pro-atherogenic phenotype [23, 24, 25]. A recent study by Chen *et al*. [26] stressed that poor glycemic control could adversely change coronary endothelial function, the early step of plaque formation and aggravate coronary atherosclerosis. Poor glycemic control, as measured by HbA1c, is an independent risk factor for CAD [27]. It is positively related to the occurrence and progression of CAD, as well as the extension of coronary atherosclerotic lesion, and worse prognosis [28, 29]. In addition, several studies have demonstrated that the presence of diabetes is associated with the features of vulnerable plaque [30]. Unfortunately, little is known about whether the aforementioned features caused by poor glycemic control were related to coronary local inflammation. An urgent rising concern is to find a rapid and simple method for accurate detection of coronary local inflammation, which would enable better stratification of cardiovascular risk, allow identification of patients at high risk for future cardiovascular events, and provide timely appropriate risk reduction strategies.

Pericoronary FAI is a CCTA-derived, novel, imaging biomarker which could noninvasively evaluate coronary artery local inflammation [15]. However, whether pericoronary FAI is associated with glucose level in ACS patients with or without diabetes is unclear. In the present study, we enrolled patients with both baseline and CCTA before undergoing coronary angiography and excluded those with a previous history of tumor, immune disease, chronic/acute infectious diseases, or statin use within 3 months which may affect systemic and local inflammatory state. Therefore, the current results largely avoid the confounding effects of inflammatory disorder on pericoronary FAI, and may reflect the impact of glycemic control on pericoronary FAI. As demonstrated in the presented study, pericoronary FAI seemed to be associated with diabetes, and this association seemed more obvious in patients with poor glycemic control. Results suggested that poor glycemic control may cause elevated coronary local inflammation, which supported the notion that poor glycemic control aggravated the progress of atherosclerosis [31, 32], and substantiated the important role of inflammatory disturbance in the development of diabetic coronary complications [33, 34]. The results also highlighted the idea that coronary inflammation might be a potential early target for preventing atherosclerosis in diabetic patients, because atherosclerosis is actually a chronic inflammatory change of the vessel wall, and often develops asymptomatically in most cases [22]. Incidentally, the mean FAI values were different in LAD, LCX, and RCA subgroups in patients with or without diabetes. This can be ascribed to the different content of adipose tissue that surrounds each epicardial artery [15].

It is also of note that poor glycemic control contributes to the elevated FAI by increasing serum inflammatory mediators. Studies have reported that as a component of metabolic syndrome, diabetes is correlated with increased plasma concentration of inflammatory mediators in the insulin resistant states in obesity [35]. However, others found that pericoronary FAI is positively associated with local inflammatory stimuli produced by the vascular wall, but not with systemic metabolic conditions such as insulin resistance [15]. In the present study, we found that the effect of poor glycemic control on serum pro-inflammatory mediators was far less obvious, although pericoronary FAI exhibited a nominal increase as well. This finding was in line with the results of previous studies that failed to show a positive correlation between serum hsCRP levels and pericoronary FAI [36]. Similarly, our previous study indicated that pericoronary FAI was driven by local inflammatory stimuli from the lesion rather than by systemic inflammatory disorders by sampling at the site of coronary stenosis lesions using aspiration catheters [16]. In addition, serum anti-inflammatory cytokines did not seem to be affected by poor glycemic control. In line with our observations, a previous meta-analysis failed to find that serum IL-10 was different in diabetic patients and controls [37]. Notably, inflammation is a broad term encompassing lots of different inflammatory pathways. Finding specific inflammatory mediators or biomarkers associated with CVD in diabetes is crucial for developing effective strategies for CVD prevention. Therefore, in a sense, pericoronary FAI may play an important role in early detection of coronary atherosclerosis risk in diabetes, whereas systemic inflammation, such as circulating hsCRP, lacks specificity for coronary inflammation.

Long-term diabetic duration was closely associated with the impairment of coronary atherosclerosis. The CARDIA Study showed that durations of diabetes and prediabetes during adulthood are independently associated with subclinical atherosclerosis in middle age [38]. A recent meta-analysis showed that a target HbA1c of between 7% and 7.7% reduces microvascular and macrovascular events in type 2 diabetes mellitus (T2DM) regardless of the duration of diabetes [39]. In the present study, results seemed to indicate that in patients with DM, the duration of diabetes was associated with increased FAI values. In patients with well-regulated DM, however, FAI values did not increase with the duration of diabetes. The results suggested that the influence of diabetes duration on FAI values may be related to poor glycemic control, highlighting the importance of glycemic control in improving coronary inflammation.

Despite the promising findings, there were several limitations to the present study. First, this study was a retrospective analysis of existing data based on a small sample size from a single center; cases in every group were different so there may be potential selection bias in this study. Second, no follow-up was performed after CCTA in this retrospective cohort, so it was not possible to determine the correlation between the impact of glycemic control on pericoronary FAI and some hard endpoints such as myocardial infarction and all-cause mortality. Third, the HbA1c value was detected at a single time point at admission, so the long-term extent of glycemic control was unknown. Since variability in the HbA1c level may impact diabetic cardiovascular complications [40], we could not rule out the possible influence of glycemic variability on pericoronary FAI. In addition, we can see a quite interesting difference in N-terminal pro brain natriuretic peptide (NT-proBNP) and low density lipoprotein (LDL) measurements between the groups; we know that some people with diabetes belong to a group with metabolic syndrome, which forms a cluster of metabolic dysregulations including insulin resistance, atherogenic dyslipidemia, central obesity, and hypertension. This may be one reason for the difference in LDL measurements between the two groups. The differences may be statistically significant with a wider sample, but it was difficult to exclude patients with metabolic syndrome or potential metabolic syndrome at the time of inclusion. Regarding the effects of diabetes on NT-ProBNP, a recent study suggested that comorbidities such as diabetes drive myocardial dysfunction and remodeling through coronary microvascular endothelial inflammation [41]. According to this theory, the difference in NT-ProBNP may be a result of coronary inflammation activation. There seemed also to be a difference in the management of DM with insulin administration. We know that DM patients with poor glycemic control are more inclined to use insulin, but the relationship between insulin and atherosclerosis is complex, insulin has several pleiotropic effects such as antiinflammatory, antithrombotic and antioxidant properties, however, insulin actions remain a subject of debate with respect to the risk of adverse CV events, which can increase in individuals exposed to high insulin doses [42]. In our study, the correlation between insulin administration and FAI value was not statistically significant (**Supplementary Table 1**).

## 5. Conclusions 

The current study indicated for the first time that quantitative assessment of pericoronary FAI might help monitor the local inflammatory activation in diabetic patients with poor glycemic control. Therefore, pericoronary FAI evaluation, as a noninvasive imaging biomarker, may play an important role in early detection of coronary atherosclerosis risk in diabetes, and allow timely application of appropriate risk-reduction strategies in patients at high risk for future cardiovascular events.

## Data Availability

The datasets used and/or analyzed during the current study are available from the corresponding author on reasonable request.

## Supplementary Material

Supplementary material associated with this article can be found, in the online version, at https://doi.org/10.31083/j.rcm2407203.
