# Supplementary material for: Impact of Glycemic Control on Coronary Inflammation Evaluated by Computed Tomography Pericoronary Fat Attenuation Index in Patients with Acute Coronary Syndrome
Source: Rev Cardiovasc Med. 2023 Jul 14;24(7):203. doi: 10.31083/j.rcm2407203 (PMC11266463; doi:10.31083/j.rcm2407203)
Supplement: Supplementary file 1 [file 2153-8174-24-7-203-s1.zip › 2153-8174-24-7-203-s1.docx]

Supplementary Table 1: Impact of insulin administration in FAI values in patients with DM

| FAI values (HU) | Patients with DM | | *p* value |
| --- | --- | --- | --- |
|  | without insulin (n=93) | with insulin (n=27) |  |
| LAD | -79.98±7.252 | -78.41±7.875 | 0.958 |
| LCX | -74.68±11.131 | -73.00±9.923 | 0.464 |
| RCA | -82.32±8.644 | -80.37±10.400 | 0.380 |

Values were presented as mean ± SD, FAI indicates fat attenuation index; and HU, Hounsfield unit.
